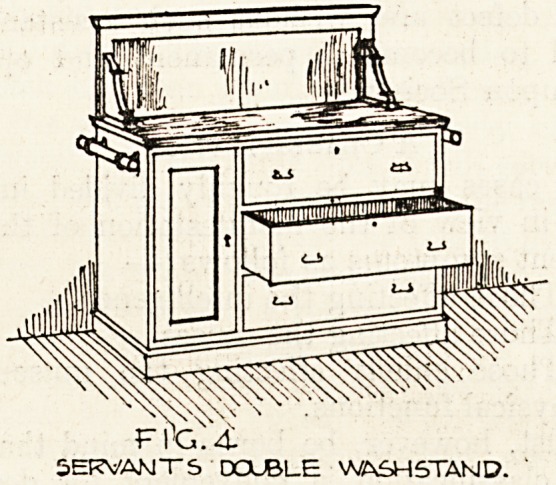# Practical Points

**Published:** 1911-11-04

**Authors:** 


### PRACTICAL POINTS.

(Criticism and Sugge tions Invited.)

### Model Furniture for Staff Use.

WE illustrate this week further examples of bedroom furniture such as would be required for the use of the residential staff. They are taken from particulars given by Mr. Carnt of the Manchester Royal Infirmary.

FIG. 1
MEDICAL STAFF DRESSING CHEST.

Fig. 1 shows the medical staff dressing-chest which is made higher than usual to give comfort in shaving. It contains three long drawers and three smaller ones. A feature of this design is the marble top; the woodwork is of American ash polished. The mirror is of the usual centre hung swing type.

FIG. 2.

MEDICAL STAFF WASH STAND

FIG. 3
NURSES COMBINED DRESSING CHEST AND WASH STAND.

Fig. 2 illustrates a washstand of the same suite; it also has a marble top and back. The lower part contains a long drawer and underneath two cupboards, one being used for books.

The nurses are provided with a fitment which is a combination of washstand and dressing chest, fig. 3. The plat whereon stands the wash basin is of marble as well as the vertical surrounds. Two long drawers are provided and one short one as well as a commode cupboard. At the end is also a stout towel rail on bronze brackets. This is also of American ash polished in the white. The medical staff and the nurses' furniture have keys which pass all the fitments of the particular room and all are again under a master key. Close on two hundred of these nurses' stands are provided.

FIG. 4

SERVANTS DOUBLE WASHSTAND.

The servants are supplied with one double washstand to three maids, fig. 4. Three long drawers are provided, each under a separate key for the different users. It was found that a marble top could be provided at a cost of 20*s*. over that of white oilcloth so that the extra initial cost gives a saving in maintenance. Two stout towel rails are provided on brass supports, and this fitment is also of American ash. The maids are provided with the usual portable type of looking-glass. All this furniture has been made by joiners in a solid and substantial manner.

## Figures and Tables

**Fig. 1 f1:**
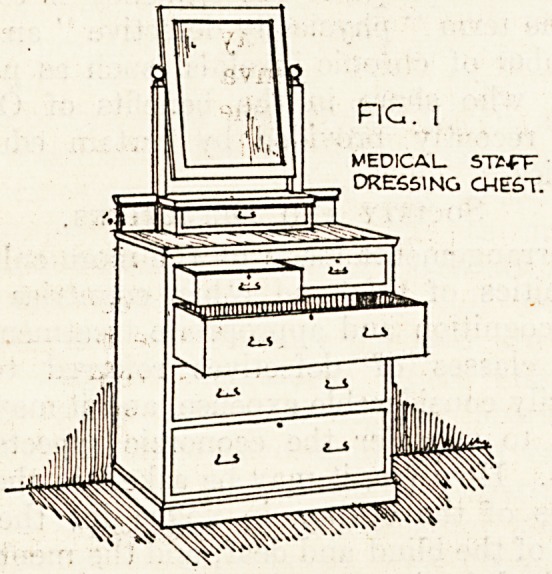


**Fig. 2. f2:**
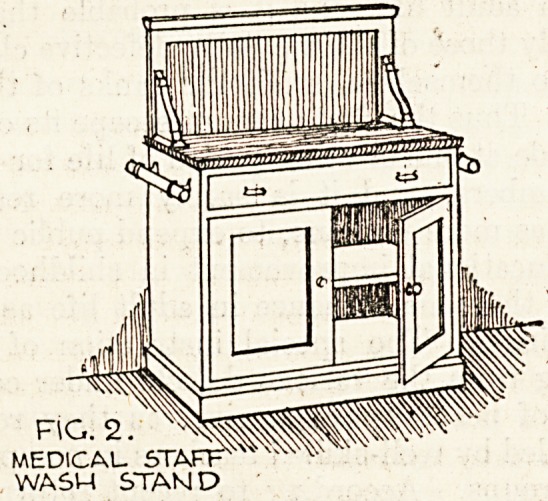


**Fig. 3 f3:**
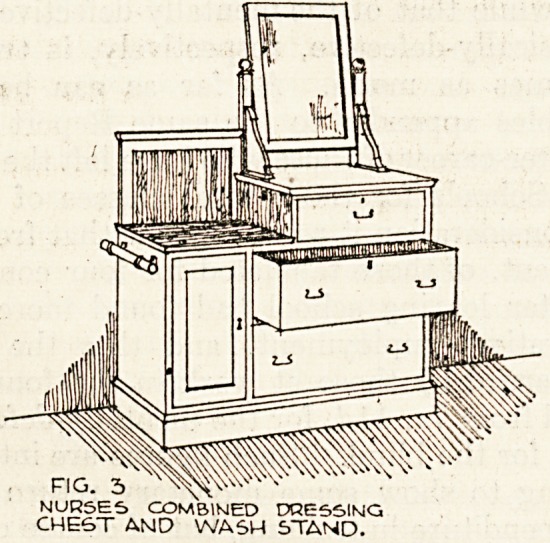


**Fig. 4 f4:**